# Retracted: Sexual Dysfunction in Women with Diabetic Kidney

**DOI:** 10.1155/2019/4390934

**Published:** 2019-12-04

**Authors:** International Journal of Endocrinology

*International Journal of Endocrinology* has retracted the article titled “Sexual Dysfunction in Women with Diabetic Kidney” [1]. The article was found to contain a substantial amount of material from previously published articles, including the following sources:

- Santoro D., Satta E., Bellinghieri G., (2014), Sexual Dysfunction in Chronic Kidney Disease, Arici M. (eds), In: Management of Chronic Kidney Disease, Springer, Berlin, Heidelberg, https://doi.org/10.1007/978-3-642-54637-2_24, [2] (not cited).
- Esposito, Katherine, Maria Ida Maiorino, and Giuseppe Bellastella, “Diabetes and sexual dysfunction: current perspectives,” Diabetes Metabolic Syndrome and Obesity Targets and Therapy, 2014, https://doi.org/10.2147/DMSO.S36455, [3] (not cited).
- Priya Anantharaman, Rebecca J. Schmidt, “Sexual Function in Chronic Kidney Disease,” Advances in Chronic Kidney Disease, 2007, https://doi.org/10.1053/j.ackd.2007.01.002, [4] (not cited).
- F. O. Finkelstein, S. Shirani, D. Wuerth, and S. H. Finkelstein, “Therapy Insight: sexual dysfunction in patients with chronic kidney disease,” Nature Clinical Practice Nephrology, vol. 3, no. 4, pp. 200–207, 2007, https://dx.doi.org/10.1038/ncpneph0438, [5] (cited as reference 6).
- R. Basson, J. Berman, A. Burnett, et al., “Report of the international consensus development conference on female sexual dysfunction: definitions and classifications,” Journal of Urology, vol. 163, no. 3, pp. 888–893, 2000, https://dx.doi.org/10.1097/00005392-200003000-00043, [6] (cited as reference 7).

The authors apologize for this. Our Editorial Board confirmed that the article should be retracted.

## References

[1] Satta E., Magno C., Galì A. (2014). Sexual dysfunction in women with diabetic kidney. *International Journal of Endocrinology*.

[2] Santoro D., Satta E., Bellinghieri G., Arici M. (2014). Sexual dysfunction in chronic kidney disease. *Management of Chronic Kidney Disease*.

[3] Esposito K., Maiorino M. I., Bellastella G. (2014). Diabetes and sexual dysfunction: current perspectives. *Diabetes Metabolic Syndrome and Obesity Targets and Therapy*.

[4] Priya A., Schmidt R. J. (2007). Sexual function in chronic kidney disease. *Advances in Chronic Kidney Disease*.

[5] Finkelstein F. O., Shirani S., Wuerth D., Finkelstein S. H. (2007). Therapy Insight: sexual dysfunction in patients with chronic kidney disease. *Nature Clinical Practice Nephrology*.

[6] Basson R., Berman J., Burnett A. (2000). Report of the international consensus development conference on female sexual dysfunction: definitions and classifications. *Journal of Urology*.

